# Strategies of a thirteen year surveillance programme on *Aedes albopictus* (*Stegomyia albopicta*) in southern Switzerland

**DOI:** 10.1186/s13071-015-0793-6

**Published:** 2015-04-09

**Authors:** Eleonora Flacio, Lukas Engeler, Mauro Tonolla, Peter Lüthy, Nicola Patocchi

**Affiliations:** Gruppo cantonale di Lavoro Zanzare - Antenna Laboratory of Applied Microbiology, Canobbio, Switzerland; Biology Institute, University of Neuchâtel, Neuchâtel, Switzerland; Laboratory of Applied Microbiology, University of applied sciences of southern Switzerland, Bellinzona, Switzerland; Microbiology Unit, Plant Biology Department, University of Geneva, Geneva, Switzerland; Microbiology Institute, ETHZ, Zurich, Switzerland; Foundation Bolle di Magadino, Magadino, Switzerland

**Keywords:** *Aedes albopictus*, Surveillance, Control measures, MALDI-TOF MS, Breeding site

## Abstract

**Background:**

In Ticino, a canton located south of the Alps in Switzerland, a surveillance programme on *Aedes albopictus* (*Stegomyia albopicta*) started in 2000 seeing that the region was considered at high risk of introduction based on the rapid spread of this mosquito in neighbouring Italy.

**Methods:**

The surveillance programme, which is still ongoing, was adapted continuously to handle preventive measures of arrival, dispersal and establishment of this invasive species. The monitoring was based on ovitraps supported by reports from the population. The integrated control measures included removal of breeding sites, larvicide applications with *Bacillus thuringiensis israelensis* or diflubenzuron and, in some circumstances, adulticide applications with permethrin. These measures involved citizens, municipalities and Civil Protection Units.

**Results:**

*Ae. albopictus* was first observed in 2003 in Ticino. We describe the strategies adopted and their adaptations to the evolving problem since year 2000. The approach used allowed keeping the mosquito densities at tolerable levels and below the threshold of autochthonous *Ae. albopictus* borne disease transmission. During the surveillance period, new typologies of breeding sites for *Ae. albopictus* have been discovered.

**Conclusions:**

It was worth tackling the arrival of *Ae. albopictus* and adopting immediate control measures, followed by regular control measures after its establishment. Early intervention and prevention of the possible spread of the tiger mosquito over the territory avoided facing a crisis situation. This also reduced the difficulty of managing the situation and probably also reduced the overall cost if this had not been put in place.

## Background

Towards the end of last century the Asian tiger mosquito, *Aedes* (Stegomyia) *albopictus* (Skuse, 1894) (Diptera: Culicidae), also known as *Stegomyia albopicta* sensu Reinert [1], was considered an invasive mosquito due to its capacity of taking advantage of global transportation of goods and traffic. This mosquito species ‘annoys’ humans because it can establish in urban settlements, bite at daytime often causing painful weals [2], and represents a sanitary risk due to its vector competence for various arboviruses and *Dirofilaria* sp. [3-5]. From its original distribution area in Southeast Asia, this species managed to spread passively worldwide throughout Africa, the Americas and Oceania mostly transported as immature stages in artificial containers, especially used tyres [6-8].

In Europe the tiger mosquito was reported for the first time in Albania in 1979 [9] and later in Italy where it spread rapidly over the country and became permanently established [10,11]. The initial detection was reported from the city of Genoa in 1990 [12] and the first evidence of its establishment was observed in the Veneto Region in 1991 [13]. *Ae. albopictus* spread in Italy was mainly promoted by the trade of used tyres containing eggs [14] and road traffic which offered passive transport over long distances for adults [15]. Within ten years, the used tyre trade, road traffic and climatic conditions facilitated the rapid spread to most regions in North and Central Italy [2]. At that time, just a few surveys started with consequent control measures on *Ae. albopictus*, i.e. in some areas of the Veneto Region [16,17], around deposits of used tyres in the Regions of Emilia-Romagna and Piedmont [18], as well as in the touristic municipality of Desenzano del Garda, in the Province of Brescia [19]. Elsewhere in Europe the tiger mosquito was detected locally in Northern France in used tyre stock, probably imported from the USA or Japan [20]. Control measures were undertaken and elimination was achieved.

In 2000, anticipating the introduction of the tiger mosquito from Italy, the mosquito working-group (Gruppo cantonale di Lavoro Zanzare, GLZ) of the Canton Ticino (located South of the Alps in Switzerland), which was in charge of controlling floodwater mosquitoes in the Plaine of Magadino since its founding in 1988 [21,22], started a surveillance program. There was no evidence yet of infestation by *Ae. albopictus* in the Italian border regions with Switzerland nor of cross-border trade of used tyres. The main risk consisted of the passive introduction of adults by road traffic since the Canton Ticino is on one of the major traffic axes connecting South and North Europe. In addition, climatic conditions in the Canton Ticino are similar to those prevailing in Northern Italy, where the tiger mosquito was at that time established [2].

The objectives of the GLZ in the surveillance strategy on *Ae. albopictus* were to keep the density of the tiger mosquito at a bearable level for residents and tourists and to prevent autochthonous transmission of *Ae. albopictus*-borne diseases as well as uncontrolled use of insecticides. In this paper, we focus on the adopted surveillance strategy and the procedure used to challenge the invasion of *Ae. albopictus* from 2000, before the arrival of the mosquito in Ticino, until 2013.

## Methods

### The study site

**Figure 1:**
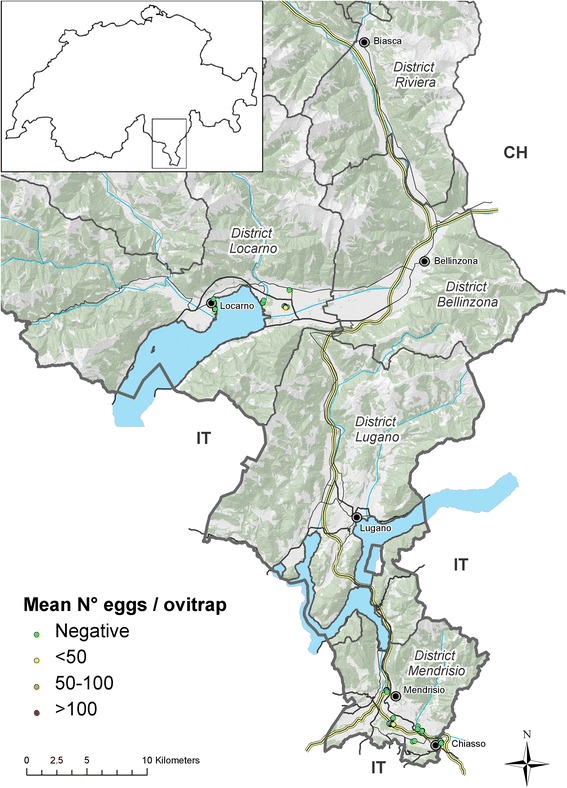
**End of the 1** ^**st**^ **period: position of ovitraps and mean number of** ***Ae. albopictus*** **eggs in 2003 in the five surveyed districts.** Most ovitraps appear as overlapped due to the low map resolution; positive ovitraps are shown at top layer.

**Figure 2:**
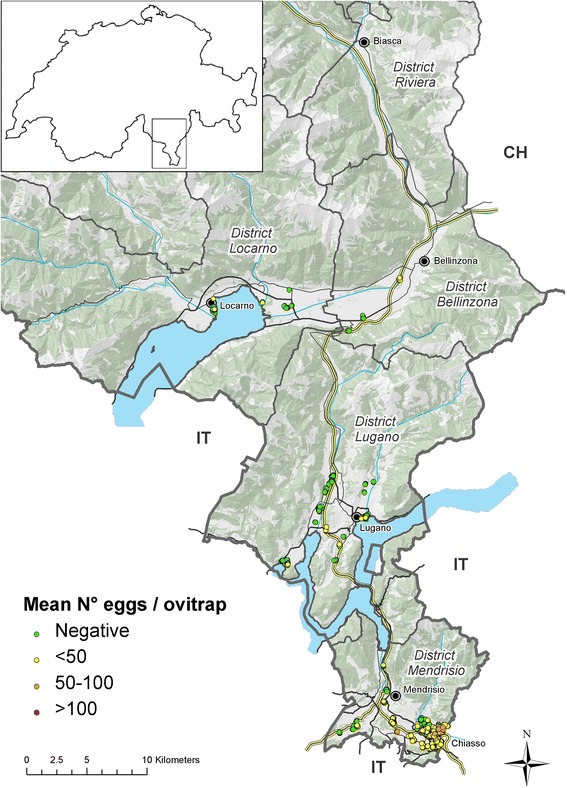
**End of the 2** ^**nd**^ **period: position of ovitraps and mean number of** ***Ae. albopictus*** **eggs in 2008 in the five surveyed districts.** Most ovitraps appear as overlapped due to the low map resolution; positive ovitraps are shown at top layer.

**Figure 3:**
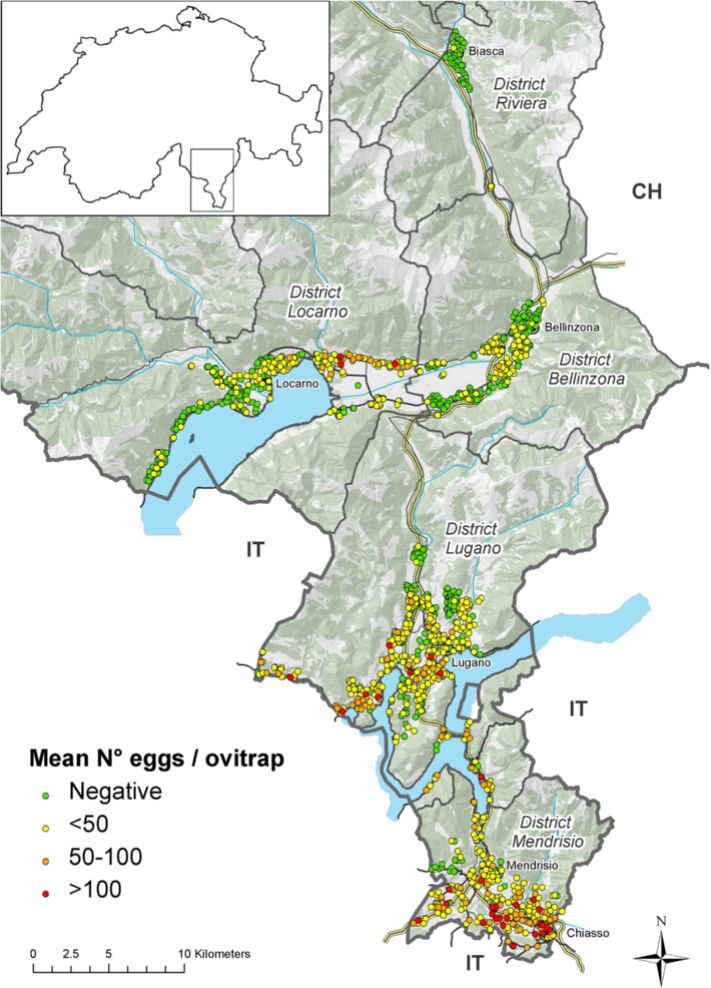
**End of the 3** ^**rd**^ **period: position of ovitraps and mean number of** ***Ae. albopictus*** **eggs in 2013 in the five surveyed districts.** Most ovitraps appear as overlapped due to the low map resolution; positive ovitraps are shown at top layer.

The Canton Ticino has a surface of 2,812 km^2^ and borders Italy to the West, South and East along 208 km. Its population is about 340,000 inhabitants and up to 60,000 cross-border workers travel daily from Italy. The Canton is divided into eight districts, five of them are included in the surveillance system: from North to South (see Figures 1, 2 and 3) 1) Riviera, (main city: Biasca); 2) Locarnese (main city: Locarno), the touristic region around the Swiss part of Lake Maggiore bordering with Italian provinces of Piedmont and Lombardy; 3) Bellinzonese (main city: Bellinzona); 4) Luganese (main city: Lugano) bordering with Italian province of Lombardy, and 5) Mendrisiotto bordering with Italian province of Lombardy (main cities: Chiasso and Mendrisio). Half of the population of the Canton lives in the Luganese district, followed by Locarnese, Mendrisiotto, Bellinzonese and Riviera (http://www3.ti.ch/DFE/DR/USTAT/index.php?fuseaction=definizioni.comuni-distretti).

The climatic conditions where the tiger mosquito was established in northern Italy and in Ticino are similar. The mean summer temperature below 400 m.a.s.l. ranges between 20°C and 22°C with an annual mean temperature of 12°C, annual precipitations in Canton Ticino exceed with 1550–1900 mm the ones in Veneto (around 600–1000 mm), whereas for the summer months the precipitations are similar, 150–200 mm compared to 100–200 mm [23,24].

### Surveillance system

The *Aedes albopictus* program, which has been fine-tuned and adjusted over the years, is based on two principal procedures: A. the survey on *Ae. albopictus*, B. the control measures that included treatments and the communication flow with the population of the Canton Ticino.

Methodologies applied during the surveillance system were based on the results obtained during surveillance activities (see results). Strategies adopted derived from what was known on the situation of the tiger mosquito at the moment, characteristics of the territory, economic resources and political interest.

The presence of *Ae. albopictus* can be divided into three successive periods. 1^st^ period: this species was not yet detected, 2^nd^ period: the mosquito appeared at several sites and had started to become established, 3^rd^ period: the tiger mosquito had colonized wide urban areas.

Some strategies remained constant during all the periods, whereas others changed or were newly applied according to the period. The adopted strategies according to periods are presented in Table 1.

**Table 1:** **General schema of the Aedes albopictus surveillance strategies and procedures adopted in Canton Ticino (Switzerland) from 2000 to 2013**

**Surveillance system**		**First period (2000-2003)**	**Second period (2004-2008)**	**Third period (2009-2013)**
Survey	survey aims	detect mosquito presence at it's arrival	intercept mosquito at arrival and analyse it's spread	analyse mosquito's spread and oversee it's densities
	surveyed areas	points of entry and development (major parking places, cemeteries, parks, etc) and resting places along the highway	points of entry and development (major parking places, cemeteries, parks, etc), industrial areas, whole municipalities (if positive), resting places and parking's along the highway	whole municipalities with grid system, resting places and parking's along the highway
	site selection for trap positioning	GLZ	GLZ	GLZ and municipalities
	traps managing	GLZ	GLZ	municipalities
	collection with other traps	no other traps used	BG-sentinel, Human Landing Collection	BG-sentinel, Human Landing Collection
	contact with residents	phone, mail and e-mail (shipping suspicious insects)	phone, mail and e-mail (shipping suspicious insects)	phone, mail and e-mail (shipping suspicious insects) and web page
	management of resident breeding sites inspection	GLZ	GLZ	GLZ and municipalities
	warm season survey period	mid-June till mid-October	April till mid-October	May till end-September
	cold season survey period	no survey	no survey	December till May (GLZ)
Control	control aims	elimination	elimination or control of densities	control of densities
	decision, responsibility and supervision of treatments	GLZ	GLZ	GLZ
	treatments timing	once positive	once positive	once positive or monthly if most of municipality was found positive
	treatments areas	around positive site	around positive site or the entire municipality (private and public areas)	the entire municipality (private and public areas)
	treatments execution	GLZ	GLZ (with help of municipality and Civil Protection)	municipality with help of Civil Protection under the supervision of GLZ
	larvicide used	*Bti*	*Bti* or diflubenzuron (for large scale treatments)	diflubenzuron or *Bti* (if requested from the municipality)
	adulticide used	permethrin	permethrin	permethrin (only in schools etc. or for epidemic risk reduction)
	removal of breeding sites	GLZ	GLZ and municipality	GLZ and municipality (ordinance)
	areas concerned for removal of breeding sites	around positive site	door-to-door	when reported
	information	media (newspapers, radio and television)	media (newspapers, radio and television), web page and specific leaflets	media (newspapers, radio, television), web page, specific leaflets and instruction on *Bti* tablets usage for citizens

GLZ: Gruppo cantonale di Lavoro Zanzare, see text.

*Bti: Bacillus thuringiensis israelensis.*

Data were organized in a relational database created with Microsoft Access, the georeference of the sampling site positions was taken from https://map.geo.admin.ch and maps were created with ESRI ArcGIS®.

### A. Survey

The survey scheme is based on the positioning of traps in carefully defined sites, analysis of detected breeding places and evaluation of reports by residents.

#### Trapping

The principal tool used for detecting the presence of *Ae. albopictus* were the oviposition traps (ovitraps) where the mosquito can lay its eggs. Ovitraps consisting of black plastic containers of 1,5 l (Ramona Ø13/H12, Luwasa® Interhydro AG, Allmendingen, Switzerland) with an efflux hole on the top border were used. A slat of steamed beech wood (200 × 25 × 5 mm) was placed as an oviposition support in an angular position in the plastic container, which was filled with tap water. Ten granules of VectoBac G® (Valent BioSciences, Libertyville, USA) were added in order to block the development of hatched larvae. The ovitraps were labelled with all the necessary information including phone number and the pledge not to remove it. Ovitraps were controlled by regular checking rounds. They were rinsed and filled with fresh water and granules. The wooden board was retrieved, labelled, wrapped into a plastic film and replaced. The boards were taken to the laboratory for examination for the presence of eggs. During the 1^st^ period, checking rounds occurred monthly except along the highway where they were checked bi-weekly. Similarly during the 2^nd^ period, in control areas where the mosquito was not yet detected checking rounds occurred monthly. Otherwise the traps were controlled bi-weekly.

For adult sampling BG-sentinel traps (Biogents® AG, Regensburg, Germany) equipped with BG-Lure (Biogents® AG) and dry ice as attractants were placed on the ground close to vegetation or buildings for 24 h. BG-sentinel traps were used occasionally for comparison with ovitraps and by biologists to estimate nuisance or to collect adults for detection of arboviruses [25]. For instant catches of adults an insect net or aspirator (Flashlight Aspirator, 2809C, www.bioquip.com) was used. To estimate nuisance, we performed human landing collections by counting the number of females landing within 15 minutes [26]. Captured adults were killed by exposure to dry ice then stored at −80°C for further virus analysis [25] or dry stored for conservation purposes.

#### Identification of mosquito species

Mosquito adults and larvae were identified using morphological keys [27,28], while eggs were morphologically identified under a dissecting microscope according to Zamburlini & Frilli [29]. Morphological identification of eggs was regularly confirmed by larvae hatched from the eggs or by using the Matrix Assisted Laser Desorption Ionization - Time Of Flight Mass Spectrometry technique (MALDI-TOF MS) [30].

#### Selection of the control locations

The selection of the areas to survey for the presence of *Ae. albopictus* was based on two key points: the potential for the site to be an entry point for the mosquitoes by road traffic and the suitability of the site for the development of *Ae. albopictus*. Ovitraps were positioned close to or under vegetation or near buildings. An important factor was to select the most suitable places to position the ovitraps: they had to catch the mosquito at its arrival place, i.e. escaping from a vehicle, before finding several other breeding sites and/or they had to be placed where adults could easily live, i.e. presence of hosts for blood meal, shadow, etc. We also took into consideration the priority of a location to be checked taking into account the nuisance and the impact of mosquitoes on sanitary and economic situations, i.e. schools, public parks, touristic places.

During the 1^st^ period there was still no record on the presence of *Ae. albopictus* in the territory. The territory was first screened with a general look on major breeding sites favourable for settlement and entry points for the mosquito, i.e.: highway resting places, parking places of the main supermarkets, deposits of used tires, main parking sites, airports, camping sites, customs, train stations, ports, recreation sites. At the same time we checked the most representative places where this species could easily develop, i.e. cemeteries, greenhouses, deposits for used tires and urban parks were inspected.

When the mosquito appeared in the territory (2^nd^ period) the survey was extended to other possible entry points, that is to say industrial areas, considered significant for the risk of introduction due to the daily traffic of goods and workers (in the case of canton Ticino, mainly cross-border workers) from Italy. In addition the survey was extended to surrounding areas where the mosquito was detected by traps, residents’ reports (see *Reporting by residents*) or personal observation of survey technicians’.

During the 3^rd^ period, when the tiger mosquito started to colonize urban areas, there was the need to have a finer net of ovitraps to closely monitor and map the dynamic of the spread of *Ae. albopictus*. The survey on the highways stops was maintained, but in addition the survey was extended to the entire urbanized areas of municipalities where the mosquito was already detected or of those municipalities considered at risk of introduction. In this case a grid system was adopted to standardize the distribution of ovitraps on the territory. The entire cantonal territory was divided into a grid of 250 × 250 m units, but only units concerning urbanized and industrial areas were considered. Each unit was designed as checkpoint where ovitraps were positioned. Since it was not possible to survey all municipalities of the Canton Ticino, there was the need of giving priority to municipalities and then selecting some of them according to the available budget. Adopted criteria and their priority scale are indicated in Table 2, whereas priorities appear in Table 3. The upper altitude limit was set at 400 m a.s.l. because the higher densities of *Ae. albopictus* in Italy were observed below this limit [31], although the tiger mosquito was also reported as high as 600 m a.s.l. Every year municipalities or new territories of a municipality (>400 m a.s.l.) were added following the same priority criteria.

**Table 2:** **Criteria and scores adopted for the choice of municipalities that were to included in the surveillance system**

**positivity of ovitraps** ^**1)**^	negative	0
	close to municipality with ovitraps positive once a year	1
	close to municipality with ovitraps positive several times a year	2
	close to municipality with ovitraps regularly positive	3
	municipality with ovitraps positive once a year	4
	municipality with ovitraps positive several times a year	5
	municipality with ovitraps regularly positive	6
**stops on the highway**	hard shoulders	1
	gas stations	2
**traffic of wares**		
commercial customs^2)^	≤ 0.1 millions/t/year of wares	3
	0.1 < x ≤ 2 millions/t/year of wares	6
	2 < x millions/t/year of wares	9
carriage free^2)^	x = number of points	3x
companies with customs control^2)^	1 company	2
	2 < x ≤ 4	4
	5 ≤ x	6
transport companies	x = number of companies	x
cargo vessels^3)^	x = number of SBB-Cargo (train cargo service)	x
*Total value = commercial customs value + carriage free value + companies with customs control value + transport companies value + cargo vessels value*		
**attraction points**		
sport centres^4)^	x ≤ 5	1
	5 < x ≤ 10	2
	10 < x ≤ 20	3
	x > 20	4
shopping centres	x = number of shopping centers	2x
tourist attractions^5)^	x = number of tourist attractions	x
train stations^6)^	regional	1
	international	2
airports^7)^	x = number of airports	x
*Total value = sport centres value + shopping centres value + tourist attractions value + train stations value + airports value*		
**cross-border workers** ^**8)**^	x = number of workers/urban settlement area (km^2^)	x
**population** ^**8)**^	x = number of inhabitants/urban settlement area (km^2^)	x
**entering cars** ^**8)**^		
customs crossing	≤ 1 million persons/year	1
	1 < x ≤ 5 millions persons/year	2
	5 < x ≤ 10 millions persons/year	3
	10 < x ≤ 60 millions persons/year	4
traffic on highway exits	≤ 20'000 cars/day	1
	20'000 < x ≤ 50'000 cars/day	2
	> 50'000 cars/day	3
*Total value = customs crossing value + traffic on highway exits value*		
**overnight stays** ^**8)**^	x = number of overnight stays/urban settlement area (km^2^)	x

^1)^survey's data (previous years).

^2)^AFD - Amministrazione federale delle dogane (Swiss custom administration).

^3)^SBB-CFF-FFS.

^4)^GIS - Geographical Information System.

^5)^Ticino turismo (Ticino tourism).

^6)^SBB-CFF-FFS.

^7)^Swiss Topo.

^8)^USTAT - Ufficio di statistica del Cantone Ticino (Statistic office Canton Ticino).

**Table 3:** **Priorities given to municipalities in joining the surveillance system**

**Index**	**High priority**	**Medium priority**	**Low priority**
positivity	>3	3	<3
stops on the highway	>1	1	0
traffic of wares	>9	4-9	<3
attraction points	>9	7-9	<6
cross-border workers	>800	500-800	<500
population	>3500	2500-3500	<2500
entering cars	>4	3-4	<3
overnight stays	>200000	100000-200000	<100000

Note: If less of 5% of municipality territory is below 400 m a.s.l. the municipality was not included in the survey. For the other municipalities a decreasing gradient of priority index going from positivity index to overnight stays index was applied.

#### Number of ovitraps in the control location

The number of ovitraps in a location, i.e. parking place, etc., depended on the characteristics of the site (e.g. different areas for car stopping, risk of manumission). Therefore several traps (up to 15) were placed in the same thematic area in order to cover all the arrival and spreading points in the 1^st^ and 2^nd^ periods. Surveillance of locations colonized by the mosquito was intensified and extended to surrounding areas once *Ae. albopictus* was discovered at the end of the 1^st^ period and during the 2^nd^ period.

With the grid system, during the 3^rd^ period, accommodating two traps every checkpoint was considered as a minimum to detect the presence of *Ae. albopictus*, based on observations made during the 2^nd^ period. Once the tiger mosquito became established in a municipality (>80% positive checkpoints) the number of ovitraps was reduced to a single one per checkpoint in order to reduce the amount of work, but maintaining the accuracy of data. The choice of the maintained ovitrap was done randomly. Hence, municipalities newly colonized by *Ae. albopictus* maintained 2 ovitraps/checkpoint, whereas municipalities with established tiger mosquito populations had one ovitrap/checkpoint. At the end of 3^rd^ period, due to budget restrictions, amount of work and wide spread of the tiger mosquito only one trap/checkpoint was positioned.

#### Period of trapping

During all periods, activities were concentrated during the warmest months that varied depending on the period (Table 1), when the probability to detect the presence of *Ae. albopictus* was the highest, according to results of Italian surveys [2] and to results obtained during the survey in Canton Ticino.

During the last two years of the 3^rd^ period, additional winter surveys were set up with 16 ovitraps positioned from December till April, controlled monthly, in the most positive sites, chosen in the most representative municipalities of two different districts.

#### Checking rounds controlled

For 1^st^ and 2^nd^ periods all the paddles of the ovitraps were controlled in the laboratory, while at the end of 3^rd^ period (2013) the analysis for the presence of eggs was done only every second checking round and the regular biweekly analysis was maintained only for one municipality/district, i.e. about every 100 km^2^. The municipality was chosen among the ones, which provided regular data and were located centrally in the district. The remaining labels were stocked for contingent further analysis, like doubts for treatment areas, risk of epidemic or studies on a specific area.

#### Identification of breeding sites

During fieldwork with ovitraps it was important to gain an overview of the potential breeding sites. Therefore places where water could accumulate had to be checked, focusing especially on those containing < 200 l [4], i.e. catch basins, plant saucers, drums, buckets, tarpaulins, tyres, bathtubs, but without neglecting the larger ones. Tools used to check these places were standard pint dippers (model 1132, BioQuip Products, Rancho Dominguez, USA) or a Pasteur pipette with the tip cut off or a fish net for aquariums. Larvae were stored in 70% ethanol whereas pupae were kept in the laboratory in plastic trays at 28°C, with 12 hours/daylight, until they emerged as adults and were identified to species.

#### Reporting by residents

The system of trapping was insufficient to cover the whole territory. Therefore community participation was organized: residents were asked to signal the occurrence of *Ae. albopictus*. Experts of the Mosquito Working Group (GLZ; see *Actors of the surveillance system*) were at disposal to answer questions and to determine insects collected by residents. Citizens were asked to send specimens to the laboratory. When the specimen was identified as *Ae. albopictus,* GLZ experts went to the field for further analysis. If a municipality outside the survey resulted positive, it was included in the survey in the following season.

### B. Control measures

Control measures consist of integrated strategies to eliminate or reduce the presence of *Ae. albopictus* including information to authorities and residents, breeding site removal and treatments.

#### Information

Urban areas in Canton Ticino are composed mostly of small private properties therefore to prevent the establishment of the tiger mosquito a targeted information campaign for the population was necessary. Mass media like TV, radio and print media were used from the beginning of the survey to inform the population on the characteristics and risks related to *Ae. albopictus* during the summer season. From the 2^nd^ period, information on the situation about the mosquito and the measures undertaken by authorities was added, and citizens were asked to avoid having domestic breeding sites. This information belonged to an annual scheduled communication flow, which started after a press communication released by the GLZ around May and was repeated in July and beginning of September in order to reach as many people as possible. In addition, a GLZ web page (www.ti.ch/zanzare) was built containing downloadable general information on *Ae. albopictus*, annual reports of GLZ, FAQ format and leaflets targeting citizens. The leaflet, available in Italian, French and German, was a key tool in the fight against *Ae. albopictus*. They contained explanations on the risks related to the tiger mosquito (nuisance and vector capacity), the potential domestic breeding sites and the measures to undertake to prevent the reproduction of the mosquito (avoid, remove or treat breeding places). In addition from the 3^rd^ period, residents where asked to treat breeding sites for *Ae. albopictus* they could not remove, therefore a list of *Bacillus thuringiensis israelensis* (*Bti*) tablets available on the market and their suggested dilution were included on the web page. In all documents phone numbers for notifications of the mosquito presence or request of information were available. Furthermore in the 3^rd^ period, municipality authorities transmitted annual information to residents in May, regarding the situation of *Ae. albopictus* presence in their territory, the measures to be taken, the dates of planned treatments, suggestions. The same information had to be present on the municipality webpage as well.

#### Removal of breeding sites

Removal of breeding sites was considered as a first priority of control measures. Residents were urged to remove them with a specific door-to-door distributed leaflet, also available on the GLZ web page. During the 2^nd^ and 3^rd^ periods (see results), in areas concerned by door-to-door treatment actions, breeding sites were removed directly by GLZ, Civil Defence Units or municipality workers after agreement with the residents. During the 3^rd^ period the GLZ suggested the municipalities to produce a specific ordinance not permitting uncared breeding sites for the tiger mosquito on the municipality territory. Therefore municipality workers were able to verify the presence of breeding sites in private domains and possibly to give penalties.

#### Treatments

Larvicides and adulticides were applied to control *Ae. albopictus*. The focus was clearly on larvicides because the immature stages were present in well-defined habitats. Furthermore the effect of larvicides was longer lasting and impacts on the environment were reduced. Larvicide applications were executed always when *Ae. albopictus* was detected. Adulticides were only carried out in parallel to larvicides when the mosquito was not yet established (end of 1^st^ and 2^nd^ periods) or when it was necessary to protect sensitive places like schools, kindergartens and senior residences (3^rd^ period).

It was decided to use two active ingredients for larvicide treatments: *Bti* or diflubenzuron and for adulticides pyrethroids like permethrin or cypermethrin. The applied products were the only ones whose use was permitted against *Ae. albopictus* in Switzerland. In fact, not all the products used are registered for mosquito control, but the GLZ received a temporary permission to use some biocides for the control of the tiger mosquito. For *Bti*, 30 granules/catch basin (average water content of 50 l water) of VectoBac G® (Valent BioSciences) were used. A concentration of 0.025 ml/catch basin was applied when the liquid formulation (Solbac®, Andermatt Biogarten AG) was applied, and 1 tablet per 20 catch basins was used for the solid formulations (Solbac-Tabs®, Andermatt Biogarten AG; Bio Garden Trauermücken-Stopp®, Migros-Genossenschafts-Bund; Coop Oecoplan Biocontrol Mücken-Tabletten®, COOP). When diflubenzuron (Device® SC-15, Paradiffusion SA) was used, 10 ml of a 2 ml/l solution were applied either with a dosage bottle (Schnelldosierflasche cat. No. 3807, Semadeni Plastics Market AG, Ostermundigen, Switzerland) or with knapsack sprayer in each catch basin (50 l). The quantity of applied insecticide was reduced proportionally if the capacity of the breeding site was smaller. Diflubenzuron was applied only if no rainfalls were forecasted for the next 3 days. During the larvicide applications all permanent breeding places, such as catch basins, were treated, whereas not permanent ones were, if possible, removed.

VectoBac G® was the only *Bti* larvicide applied in not extensive treatments during the end of the 1^st^ period and the 2^nd^ period, whereas during treatments on large surfaces at the end of the 2^nd^ period and during the 3^rd^ period a liquid formulation was preferred and a longer lasting insecticide was necessary in order to avoid frequent applications. The IGR diflubenzuron was introduced, which permitted application lasting for about one month [32,33]. *Bti* liquid formulation (Solbac®) was used only in case of unfavourable weather forecast to replace diflubenzuron in already planned larvicide applications or in case a municipality expressed the wish not to apply IGR. In this last case, application on catch basin had to be repeated every week, because the effect of used *Bti* formulation is immediate and has no persistence. The insecticide application had to interrupt the mosquito aquatic phase that, during summer climatic condition lasts about a week. *Bti* tablets were used by residents to treat their domestic breeding sites, with the recommendation to apply this insecticide once a week, from May till the end of September.

Adulticide applications were executed during the evening hours on the vegetation surrounding positive locations up to 2 m above the ground level with a backpack sprayer using permethrin (Matil®, Maag AG) or cypermethrin (Cypermethrin®, Sintagro AG), following dilutions recommended by the manufactures. Applications have to hit the target with limited side effects on non-target organisms, therefore: in order not to affect their waters, adulticide treatments were not performed close to rivers or lakes, in addition grasslands were barbed before, so they did not need to be treated and edible vegetation was not concerned to avoid any health problem to the population.

The adulticide and larvicide applications were performed on a radius of 100 m and 200 m around positive traps respectively, shortly after the detection of *Ae. albopictus*, i.e. in the 2–4 weeks following detection in order to eradicate the mosquitoes when the tiger mosquito was not yet widely established (1^st^ and 2^nd^ periods). Large surfaces applications using both larvicide and adulticide applications were performed only at the end of the 2^nd^ period. In this case, all treatments were performed door-to-door and the population was informed through media (television, radio and newspapers) as well as through a leaflet in their letterbox. Treatments occurred twice in the warm season: around the beginning of June and between the end of August and the beginning of September. During the 3^rd^ period, when *Ae. albopictus* was already widespread, the treatment goal was no longer the mosquito elimination but rather the control of its density. Treatments were planned already during wintertime. Three main seasonal interventions were planned on the territory in accordance with the availability of the regional Civil Protection members (see *Actors of the surveillance system*): a) between mid-May and mid-June, b) in July (not always possible) and c) between mid-August and mid-September. The first treatments aimed at targeting the first juvenile stages, the second to control densities before the summer peak of August-September, while the third aimed to reduce the adult population laying overwintering eggs. The surface to be treated for the first intervention was based on data from the survey of September of the previous year, while the treated surfaces for the second and third interventions fitted on data from the on-going survey. Until 2013 (end of the 3^rd^ period), interventions were lead in public and private domains, i.e. treating catch basins on public streets and treating or removing door-to-door every breeding site, while since 2013 applications concentrated only on public areas. Municipalities infested by *Ae. albopictus* were asked to apply larvicides on a monthly (diflubenzuron) or weekly (*Bti*) basis in public areas from May till October. Concomitantly citizens were asked (through specific information leaflets) to treat their private property with *Bti* tablets.

#### Actors of the surveillance system

The pre-existing Mosquito Working Group (GLZ) took the responsibility to start the surveillance system on *Ae. albopictus* among its general duties on mosquitoes. By the end of the 2^nd^ period, the GLZ was officially in charge of the surveillance on *Ae. albopictus* and a specific group was added to face problematic connected with the presence of the tiger mosquito. This group included cantonal representatives of health, tourism, territory, environment, veterinary institutions and concerned municipalities.

All the morphological determination were lead by specialists of the GLZ for all the three periods, whereas survey and control measures in the 3^rd^ period by the increasing of the presence of the tiger mosquito in the territory were actuated by different actors: GLZ, municipality workers and Civil Protection Units.

For the 1^st^ and 2^nd^ periods all the survey was lead by a biologist of the GLZ employed 50% for the 1^st^ period and 100% for the 2^nd^. Treatments were planned and carried out by the biologist with the help of 2 workers of the GLZ. During the last year of the 2^nd^ period treatments on large surfaces in concerned municipalities were started. In this case workers and soldiers of the Civil Protection were coordinated by the biologist of the GLZ, with the help of municipality. All kind of treatments was performed under the supervision and organisation of GLZ workers patented for use of biocides.

For the 3^rd^ period two work units (80% each) were employed during the year and 4 other work units (50% each) for the six warm months were added.

During the 3^rd^ period the surveillance work on *Ae. albopictus* obtained the collaboration of the concerned municipalities. According to the health care law in the Canton of Ticino, the municipalities have to take measures to fight against organisms that transmit diseases on their territory, and GLZ acted as consultant and coordinator of the activities. Therefore a constant communication flow was established between municipality authorities and GLZ. Each year, in January, an annual report and a report specific for each municipality were sent. In addition, between the end of March and mid of April, all municipality delegates were invited at annual meetings with representatives of regional Civil Defences in order to discuss and adjust methodologies and strategies as well as the time schedule for control measures. During the surveillance season the GLZ was at disposal of the municipality authorities for any kind of problems and all communications from citizens concerning *Ae. albopictus*, which were supervised by the GLZ.

Technicians of the surveillance programme together with municipality delegates selected the places for ovitraps. Personnel of each municipality carried out the routine check of ovitraps while the counting of eggs on the collected wooden paddles was done by the GLZ personnel. Therefore in 2009 the GLZ requested the active participation in the survey and control measures of municipalities considered at major risk. The grid system applied on the territory and the planned treatments permitted to rationalize and delegate part of the work to municipality personnel, while the GLZ maintained the supervision of the whole process.

In all surveys, the highway was included as well, but surveyed and controlled by GLZ personnel only.

Civil Protection joined regularly the municipalities in the treatment actions, which were planned already during wintertime. Three main seasonal interventions were planned on the territory in accordance with the availability of the regional Civil Protection members.

## Results

In this paper the results are described and divided into three successive periods according to the presence of *Ae. albopictus* in the territory (see *materials and methods*). The surveillance results obtained during these successive periods were the basis for decision for further measures, that is to say they were the basis for the different methodologies applied during different periods and described in the materials and methods section.

### 1^st^ period (2000–2004)

Being aware of the high risk of passive introduction, we initiated a proactive monitoring of *Ae. albopictus* in 2000. At that time, there was no sign of the presence of *Ae. albopictus*, but there was a high risk of passive introduction through road traffic from Italy. Therefore surveillance was mainly focused on possible points of entry at the state border and along the South–North highway (E35), which is the only highway in the Canton. At the same time the most representative places where this species could easily develop, i.e. cemeteries, greenhouses, deposits for used tires and urban parks were inspected.

In year 2000, the territory was first screened with a general look on major breeding sites favourable for settlement and entry points from Italy. Since *Ae. albopictus* was not observed, some checking points were left aside the following years, because they did not appear as a priority. In fact, deposits of used tires had no trade with foreign countries, cemeteries were mostly very dry and greenhouses did not represent a main place for traffic stop. The survey was then extended to other main traffic parking places.

During this start monitoring period used tires were also adopted as traps, but their managing was difficult compared to ovitraps and this method was not further used.

In 2003, a total of 34 ovitraps were positioned at the beginning of July until mid-September (Figure 1). Principally 6 bi-weekly checking rounds were conducted but some ovitraps were controlled just monthly. Hence a total of 166 ovitraps were analyzed for egg presence on their slats. Seven ovitraps turned to be positive. In fact *Ae. albopictus* was found for the first time between mid-July and end-August 2003 in Switzerland in four ovitraps, in the Mendrisiotto, at the first service station on the highway entering Switzerland from Italy (Figure 1). Similarly three ovitraps were found positive between end-August and mid-September in a small airport close to Lake Maggiore, in the Locarnese district. These ovitraps had a mean number of eggs <50. After these observations, the number of ovitraps was doubled in order to improve the detection for the following checking rounds.

Larvicide and adulticide applications were performed after the discovery of *Ae. albopictus*, end-August on the highway and mid-September in the airport. No *Ae. albopictus* was found after the treatments till the end of the surveyed season, i.e. mid-October 2003.

In the following year traps were set on the northwards service station on the highway and at the Lugano-Agno airport, in the Luganese district. A total of 57 traps were set, resulting in 465 checks during the season, of which 23 resulted positive to *Ae. albopictus*. The presence of the mosquito was confirmed at the small airport of Magadino, in the Locarnese district and in the first service station on the highway coming from Italy in the Mendrisiotto district. Additionally the mosquito was found in the second service station on the highway and in a cargo train station in the Mendrisiotto district. Again control measures were undertaken.

### 2^nd^ period (2005–2008)

Following *Ae. albopictus* discovery in Canton Ticino in 2003, and confirmation of its presence in 2004 on the highway and in one airport (Magadino), as well as its appearance in the cargo train station, it was decided to implement the survey on other possible entry points. Hence the survey was extended to the industrial areas, where most of the cross-border workers coming from Italy stop, and to houses surrounding the service stations along the highway, when they were found positive.

During the 2^nd^ period, *Ae. albopictus* was detected sporadically at several locations and started to settle in the territory (Figure 2). Therefore, the survey was extended to additional industrial areas, to the urban areas of positive municipalities and in all resting places along the highway.

In 2007 the town of Chiasso, at the border with Italy was found positive thanks to a report from a citizen by the end of September 2006. In 2008, two additional municipalities of Mendrisiotto district close to that border area, Morbio Inferiore and Vacallo, were included in the surveillance system. In 2008, tiger mosquitoes flying directly across the border from the Italian municipality of Maslianico (Province of Como) to Vacallo were observed and most of the inspected catch basins in the Italian municipality (Maslianico) were found positive to *Ae. albopictus* (Dr Romeo Bellini and Eleonora Flacio, personal observations).

At the end of the 2^nd^ period, in 2008, 466 ovitraps were positioned from the beginning of April to mid-October (Figure 2). A total of 4,919 controls of ovitraps were carried out in 14 bi-weekly checking rounds and 436 turned to be positive. *Ae. albopictus* was prevalently detected in Mendrisiotto, but it appeared as well in the Luganese and Locarnese districts, and the ovitraps at the resting places along the highway started to be constantly positive to the mosquito. The average number of eggs/season varied but did not reach more than 100, the highest being in the southern part of the studied area. The first service station on the highway in Mendrisiotto was still positive, whereas the airport close to Lake Maggiore not.

Until 2007 larvicide applications were performed by GLZ workers with VectoBac G® and continued on highway resting places until the end of the 2008. In July 2008, we started applications on large surfaces with the help of municipality workers and soldiers of the Civil Protection therefore a longer lasting insecticide was necessary in order to avoid frequent applications, thus the IGR diflubenzuron was introduced. If the positive areas were restricted, treatments managed generally to eliminate the mosquito. One such example is the main park in Lugano, Parco Ciani, where the tiger mosquito was found for the first time in July 2005, disappeared after the treatment and was found again only at the end of June 2008. Similarly to the 1^rst^ period, treatments were carried out shortly after the detection of *Ae. albopictus*, i.e. in the 2–4 weeks following mosquito detection in order to eradicate the mosquito or control its density.

### 3^rd^ period (2009-up to now)

During the 3^rd^ period, when the tiger mosquito started to spread in the territory, municipality authorities were directly involved in the survey from the beginning of the colonization of the tiger mosquito. The grid system to standardize the distribution of ovitraps on the territory was adopted in the municipalities involved in the surveillance program. The active requested participation of the municipalities for the survey as well as for the control measures allowed from the beginning to radically increase the surveyed area and gave a better information accuracy regarding the presence of the tiger mosquito. The number of traps set was raised from 466 in 2008 to 1,241 in 2009, the number of controls on ovitraps from 4,919 to 10,526 and the number of involved municipalities increased from 21 in 2008, (when the entire urban settlement was not yet considered and no grid system was in place) to 37 in 2009 when the entire urban settlement with the grid system was arranged. In 2013 the number of involved municipalities was 61, covering 76.5% of the population of the Canton Ticino.

During the last two years of the 3^rd^ period, additional winter surveys were set up with 16 ovitraps positioned from December till April, controlled monthly, in the most positive sites, Chiasso and Lugano belonging to Mendrisiotto and Luganese, respectively (Figure 3). However, the winter surveys did not show any activity of *Ae. albopictus* in the ovitraps. Active *Ae. albopictus* adults were found from mid-May to beginning of June until mid-November.

*Ae. albopictus* started to be found in large urban areas along the southern border with Italy and more frequently in cities like Lugano and Locarno (Figure 3).

The screening of the ovitrap paddles for mosquito eggs by the GLZ became more and more time-consuming (up to 13,418 paddles analyzed in 2010), therefore a reduction of the number of ovitraps was applied for municipalities with >80% of positive ovitraps over the season. In addition a reduction of checking rounds analyzed for eggs was applied in 2013 (see *methods*). During the 2013 survey (Figure 3), 1,389 ovitraps were positioned in the field. A total of 7,069 controls of ovitraps were carried out among the checking rounds analyzed during the season and 2,081 turned to be positive. Positive ovitraps appeared all along the highway. The average number of eggs/season increased and average densities of more than 100 eggs per ovitrap during the season were observed at various locations, whereas in most surveyed locations densities used to be lower. The mosquito was widespread in most regions, except in the Riviera district where only one or two traps were positive to *Ae. albopictus* during the survey season. In the Mendrisiotto district, where the mosquito appeared first in 2003 (Figure 2), almost all ovitraps were positive, but high egg densities were present mainly around the service stations on the highway and along the border with Italy. The airport close to Lake Maggiore remained negative (Figure 3).

**Figure 4:**
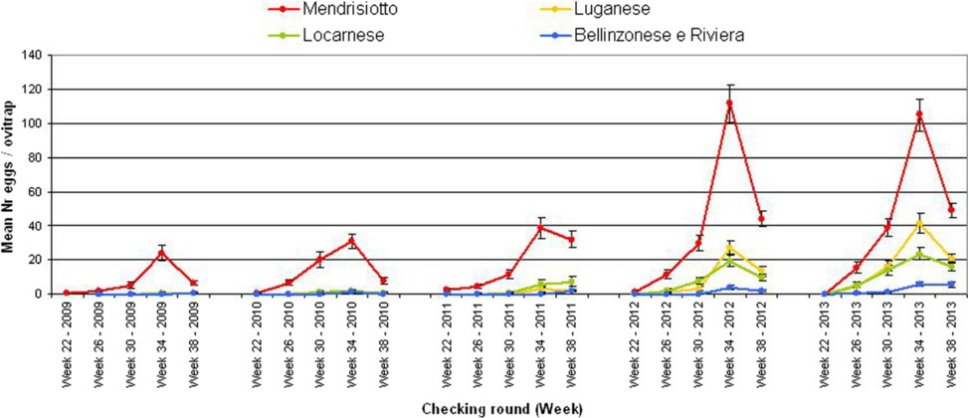
**Seasonal trend of the mean number of** ***Ae*** **.** ***albopictus*** **eggs in the five surveyed districts (2009–2013).** Based on data of the ovitraps placed in municipalities.

Figure 4 shows the seasonal trend of the mean number for *Ae. albopictus* eggs subdivided into the five districts of the municipality survey. A maximum of around 110 eggs/ovitrap was observed during the peak of activity. The district most concerned by *Ae. albopictus* during the 3^rd^ period was Mendrisiotto, the southern region of Ticino. In 2012 and 2013 the Luganese and Locarnese districts reached egg densities, which were previously observed in Mendrisiotto only. In Bellinzonese and Riviera, the mosquito densities increased but remained relatively low. Between 2011 and 2012 there was a prominent increase in egg densities, which remained constant during summer 2013.

#### Citizen reports on the presence of Ae. albopictus outside the surveyed area

The survey was based on results of ovitraps as well as reports of citizens who called or sent emails to the GLZ to communicate the suspected presence of *Ae. albopictus*. The GLZ received around 500 reports/year. The reports usually came from areas where the presence of the tiger mosquito was already known, but every year 1 or 2 reports allowed confirmation of the presence of *Ae. albopictus* outside the survey system. Residents contacted the GLZ not only for suspected cases, but also for any counselling regarding the tiger mosquito, i.e. treatments within their property, nuisance, risk of diseases, etc. About 35% of the contacts where phone calls, 35% were emails, while 10% were made directly to the operators of GLZ during their field work and about 20% were letters containing insects arriving by post. Feedback from GLZ were given within two weeks. Usually reports arrived from municipalities having lower priority, which had not been included in the survey because of limited financial resources.

#### New breeding sites

Inspected breeding sites corresponded mostly to what was known from the literature [4] and other survey systems, i.e. containers with < 200 l of water, but also bigger breeding sites such as oil sorters close to the highway and in large parking places could be detected. These places contained thousands of litres of water and included several connected underground tanks, close to the surface, with small openings in the manhole covers for inspection. All the other inspected oil sorters not closed at the surface were negative for *Ae. albopictus*. Larvae of the tiger mosquitoes were also found in flooded basements of building yards and in other receptacles containing thousands of litres of water, such as tanks, broken fountains, cisterns. All these large water containers were situated in shadow under vegetation.

Adults were detected in wooden areas, surrounding colonized villages, but no breeding sites could be detected: neither tree holes containing water nor general waste that could act as breeding sites [34].

#### Nuisance

In response to citizen phone calls claiming a nuisance caused by *Ae. albopictus*, GLZ workers placed BG-sentinel traps around the affected houses. For example, a total of 821 females were caught between end-August and mid-September 2013 during 37 sampling sessions, corresponding to an average of 22 females/day. Nuisance prompted citizens to adopt control measures suggested by GLZ, i.e. most of them started to remove breeding sites after experiencing the nuisance due to tiger mosquitoes.

#### Egg identification

An extensive survey applying the hatching technique on all positive paddles of the ovitraps is too time-consuming, therefore hatching was only used sporadically. In particular, hatching was performed during the 2^nd^ period, for distinguishing morphologically, under a dissecting microscope, eggs of *Ae. albopictus* from the ones of *Ae. geniculatus*, a species frequently laying its eggs on the wooden paddles of the ovitrap [29]. In 2013, eggs identified morphologically as *Ae. geniculatus* were confirmed using MALDI-TOF MS technique [30]. Among 325 samples morphologically identified as *Ae. geniculatus* only one was shown to belong to *Ae. albopictus*. In addition, for the eggs identified as *Ae. albopictus,* 3 to 5 positive traps, randomly chosen for each municipality were checked three times during the season. Samples came from 46 municipalities among 61. A total of 3,334 eggs were analyzed and all were confirmed as *Ae. albopictus* with the Mass Spectrometry technique.

## Discussion

Considering the rapid spread of *Ae. albopictus* in Italy, the GLZ realized that the Canton Ticino became a region at high risk of invasion. Therefore, a preventive surveillance system was implemented in 2000 in order to face the problem from the beginning. Continuous introduction followed by a local diffusion prompted the GLZ to adopt a dynamic and flexible surveillance system, which can be resumed in three major periods of time corresponding to the level of the spread of the tiger mosquito. Those periods fit to the three scenarios described in the recent published guidelines of the European Centre for Disease Prevention and Control [35] and the strategies we adopted from 2000 to 2013 are similar to the ones indicated in the ECDC guidelines and in the ones of WHO/EMCA [35,36].

The surveillance system, which is still ongoing, allowed the first detection of *Ae. albopictus* in Canton Ticino in 2003 [37] and the management of its spread [38-40]. Ovitraps are the main tool for monitoring species belonging to the subgenus *Stegomyia*, i.e. *Ae. aegypti* (Linnaeus, 1862) and *Ae. albopictus* [26,41]. Ovitraps detect mosquitoes even at low densities, are not expensive and are easy to handle even by unskilled staff. To be used in a large-scale survey a compromise between attractiveness for the mosquito (colour, shape, odours, persistence of water), safeness, i.e. not being breeding sites themselves, simplicity in handling and availability on the market has to be found. In fact other trap methodologies were tried, such as the trap for adults BG-sentinel (Biogents AG, Regensburg, Germany) when the tiger mosquito started to be detected in urban areas in 2008. BG-sentinel traps equipped with BG-Lure and without dry ice, were positioned at the border with Italy in parallel to ovitraps, i.e. controlled biweekly. BG-sentinel traps were positioned in gardens where the presence of *Ae. albopictus* had been detected by ovitraps or reported by residents. The average number of eggs in 10 ovitraps present in a radius of 50 meters around the BG sentinel was then compared with the number of adults in the BG-sentinels. It resulted in low densities of eggs, i.e. between 15 and 50 eggs, BG- sentinels contained 0–2 adults of the tiger mosquito. Seeing the difference in price, managing and the risk of manumission between ovitraps and BG-sentinels, the latter were abandoned for presence/absence purposes at low densities in the survey. During the same period sticky traps were tried out, but they were more difficult to operate. Ovitraps with a piece of polystyrene were tested as well. In this case, hatching of eggs was observed on it because of water capillarity, and determination of eggs was considered easier on the wooden stick in a large-scale system. Following these observations, ovitraps with the wooden paddle remained the main tool for the extensive survey system.

Whether the number of eggs present on ovitraps is correlated to the real presence of *Ae. albopictus* adults in a territory is often under discussion [41] but eggs on ovitraps can describe the presence of this mosquito correctly. In addition ovitraps are more reliable than other traps, such the adult trap BG-sentinel, to detect the mosquito presence at low densities as described above. The reduced cost in producing and checking ovitraps, permits the use of numerous traps in the survey. Even if cost-benefit at low densities of mosquito favours the use of ovitraps, a comparison with adults catches and number of breeding sites should be applied in a survey system. The density of eggs in ovitraps gives an indication of relative mosquito densities in an area compared to another one, which may represent an important indication of a problem related to the integrated control measures. Often neighbouring municipalities with the mosquito present over their whole territory show different egg averages on their ovitraps. This is an indication for the GLZ that there was a problem, such as treatments in catch basins not done with the planned schedule or bad information to residents in the municipality with higher densities. Despite these limits, mean number of eggs on ovitraps is still extensively used in surveillance systems.

*Ae. albopictus* spread could not be stopped, but adopted control measures managed to control or reduce the densities of the mosquito. The direct effect of a single type of control action was difficult to estimate, because in the system no control sites (untreated sites) were foreseen in non-managed municipalities. Usually if the mosquito disturbed the population the municipality and the citizens themselves reacted spontaneously adopting the suggested control measures. One example is the municipality of Melano (Mendrisiotto district), where the maximum egg densities of the season, i.e. end-August, increased from 65 ± 15 eggs/ovitrap in 2010, to 131 ± 30 in 2011, to 133 ± 32 in 2012 but decreased to 71 ± 23 in 2013.

The active participation of the population in managing breeding sites, joined with regular treatments of public catch basins are the key measures to control *Ae. albopictus*. This could be further improved in preparing specific educational programmes addressed to schools. For example in the surveillance system of Catalonia (Spain) a strong weight is given in educating the younger generations on the biology and breeding sites of *Ae. albopictus* (http://elmosquitigrealescola.blogspot.com.es/; http://www.mosquitigregirona.cat). This strategy, that involves children and their relatives, may engage the population deeply and sustainably in prevention. *Ae. albopictus* is a very annoying insect, biting at day-time and repeatedly, for this reason residents usually took into consideration the control measures suggested by the GLZ just after being directly concerned by the nuisance. Most of the contact to the GLZ came from residents suffering from the presence of the tiger mosquito. Citizens can obtain information on *Ae. albopictus* through the media and there is a web page dedicated to the thematic, but still the personal contact with an expert able to explain the particular case is considered important by the residents. Contact via email is rapid, however, a lot of people, in particular elderly individuals, prefer phone calls in order to delve into the subject. This service offered by the GLZ is time consuming, but has a durable effect and brings appreciation for the entire work. Mosquito densities detected in the BG-sentinel traps after phone calls to the GLZ in the peak season are similar to the ones reported in Italy [42], but in Ticino traps were positioned around houses of residents complaining about nuisance and not randomly on a surveyed area.

The implementation of the survey on tiger mosquito (3^rd^ period) showed that its seasonal activity in Canton Ticino fits with what has been observed so far in Northern Italy, e.g. on http://www.zanzaratigreonline.it. In fact there was no activity in the winter period, the mosquito breeds in urbanized areas and the choice of the municipalities to be involved in the surveillance system corresponds with citizen reports.

In general, it is important to keep the control on the typology of breeding sites to be effective in preventing and controlling the establishment of the tiger mosquito. In fact new types of breeding sites can be discovered. This is what happened for covered oil sorters and flooded basements, as for tanks, broken fountains and cisterns. Because of the large amounts of water (>200 l) they had not been considered so far as breeding sites for *Ae. albopictus*: in fact, control of breeding sites usually concentrates on small water containers [35,36]. Tiger mosquitoes seem to breed in such containers if their surface is covered either by the structure of the container itself or by vegetation. Our new observations of larger breeding sites should encourage the control of larger containers in the future.

The strategies adopted since 2000 allowed the stabilization of the system on a large scale and made the public gradually aware of its responsibility and capacity to fight *Ae. albopictus*. Our surveillance program took into account treatments of every place where *Ae. albopictus* was detected as well as the supply of information to all citizens on prevention and control measures. This is the reason why no area can act as control (untreated area) for the evaluation of our prevention and control measures. In an on-going survey, ovitraps were placed in the area of the Canton Ticino bordering with Italy and in Italian municipalities just over the border were no control measures are undertaken. First results show that densities of eggs on ovitraps are far lower in Ticino (Tobias Suter, personal communication).

*Bti* was chosen as larvicide because this microbial insecticide is selective for mosquitoes with none or light reported effect on non-target organisms [43,44]. *Bti* is therefore considered the most environmentally friendly larvicide, which is important in case of application by non professionals, such as residents or in case of difficult weather conditions (i.e. rain forecast). Limits of the use of *Bti* for extensive application are that it is not persistent and that it does not kill all juvenile stages of mosquito. On the other hand, applications of diflubenzuron, an IGR (Insect Growth Regulator) which inhibits synthesis and deposition of chitin and is characterized by a low acute toxicity against mammals and a good safety margin with respect to non-target organisms including fish and birds [45,46], have to be executed following correct dilutions and weather forecasts not to risk undesired contamination of water systems. Advantages of diflubenzuron are its persistence for about one month [32,33] and its effect on all juvenile stages of mosquitoes. Therefore treatments of catch basins during the summer season with *Bti* had to be repeated weekly, in order to interrupt the aquatic phase in day summer temperature of about 25°C, whereas diflubenzuron treatment occurred monthly. As adulticides, the pyrethroids permethrin and cypermethrin were used. These neurotoxins are more toxic against cold-blooded than against warm-blooded animals. Pyrethroids were used because of their knockdown effect (killed immediately, without sub lethal effects) and their persistence was estimated at about a few days in summer conditions. The insecticides for larvicide applications were restricted to *Bti* and diflubenzuron, because they are the only ones for mosquito control currently allowed on the Swiss territory. In the future, if registered in Switzerland, we might also apply Vectomax® (Valent BioSciences), a combination of *Bti* and *Lysinibacillus sphaericus*, which increases the persistence of the larvicide activity compared to *Bti* alone also against *Ae. albopictus* according to the manufacturer and would minimize impact on the environment in case of misuse. In any case in Canton Ticino all the applications are under the supervision of the cantonal authority for protection of air, soil and water (Sezione della protezione dell’aria dell’acqua e del suolo, www.ti.ch/spaas).

The methodology adopted in Canton Ticino for the 1^st^ and 2^nd^ period is described in the Guidelines for surveillance in Switzerland on *Ae. albopictus* available, in French and Italian, on the web pages of the Federal Office of Public Health and on the one for the Environment [47]. In fact this methodology of the 1^st^ period is the one currently applied, since 2013, for the monitoring of *Ae. albopictus* at a national level in Switzerland. This strategy allowed the recent detection of *Ae. albopictus* in 3 service stations North of the Alps along highways: 1) the service station “Gotthard”, the first service station after the Gotthard tunnel on the highway coming from Canton Ticino, 2) the service station “Heidiland” on the highway leading to Austria and Germany and 3) the service station “Grauholz” on the highway crossing Switzerland from east westwards [48-51]. The GLZ applied the collected data to a model of suitability for the presence of the tiger mosquito in order to evaluate the risk of expansion in Switzerland [50]. This model indicated that North of the Alps on the Swiss Plateau, the region between Geneva to Basel, offered favourable climatic conditions for the settlement of the mosquito. Since *Ae. albopictus* was found close to Geneva in the frame of the French “Plan National anti-dissémination de la dengue et du chikungunya” [51,52] in 2012 and 2013 as well as close to the Swiss border in three locations on the highway in Germany in 2012 [53]. The GLZ currently provides assistance to local authorities in Geneva Canton to survey *Ae. albopictus*.

Cost factors may affect strategies applied for the surveillance and the control of the tiger mosquito [35,36]. In our case, the limited budget resources for salaries and materials, which reached at the beginning around 10,000 CHF/year and rose up to 8 folds in the 2^nd^ period to 20 folds in the 3^rd^ one, as well as the increasing amount of work, due to the spread of the tiger mosquito, prompted us to constantly rationalize the system and to outsource part of the work to the concerned municipalities during the 3^rd^ period. Hence, during this period, despite the fact that we had to reduce the number of ovitraps and checking rounds, the strategy used allowed to keep an accurate vision of the densities on the territory facing the problem of the time-consuming and expensive reading of ovitrap labels.

Here, most mosquito samples were identified using morphological characteristics. The use of the MALDI-TOF MS technique [30] validated the identifications.

*Ae. albopictus* is now widely distributed, spread mainly through the used tyre trade. Its presence has been reported in the United States of America, Latin America, several Pacific and Indian Ocean islands and Europe [54]. *Ae. albopictus* has been reported in at least 11 countries in Europe [55-58]. Almost every European country has at least one part of its territory surveyed for *Ae. albopictus*. In some cases only survey strategies are applied, in other countries, control measures are foreseen in case of an epidemic of mosquito-borne pathogens, and only few systems include systematic and coordinated control measures [57,58]. The noteworthy feature of the surveillance system described in this paper is the start in 2000 before *Ae. albopictus* appeared in the territory (which happened between 2003 and 2008) and has been maintained along the whole process of the invasion of the exotic mosquito. The Canton Ticino is a small region therefore covering the territory with a surveillance system was feasible, but it is also one of the regions in Europe where the pressure for introduction of the tiger mosquito was the highest, particularly because of the intense road traffic entering from infested areas of southern Europe. In addition, climatic conditions in this canton were favourable for the establishment of the mosquito. The dispersal of the tiger mosquito was inevitable, but eliminations of new foci during its first arrival (2^nd^ period) were successful, which delayed its establishment (3^rd^ period) and even then control measures had an effect on mosquito densities. The fact, that control measures (applications and choice of insecticides) were under the supervision of a centralised authority (here the GLZ) helped to keep appropriate procedures under control. We achieved all those results by constructing the surveillance system step by step, financial restrictions and constantly increasing work prompted us to reconsider and refine strategies every year. Usually in Northern Italy the colonization process was faster with the species spreading into the whole urbanized area in 3-4 years from the first detection (Dr Romeo Bellini personal communication). Facing the problem from its beginning and maintaining trained and qualified personnel permitted us to gain knowledge of the territory and the related problematic. In addition, and this is an important point, this situation gave the population time to become aware of the *Ae. albopictus* thematic and the control measures to adopt. Before the arrival of *Ae. albopictus* in the Canton Ticino, residents were not familiar with mosquito nuisance, but with time they became familiar with the problematic related to the tiger mosquito without panic or alarmism and without consequences on the touristic activity, which is economically important for the region. We are convinced that it was worth facing *Ae. albopictus* on its arrival and adopting immediate control measures, followed by regular control measures after its establishment. In fact, this strategy permitted avoiding the surprise effect of having the tiger mosquito spread all over the territory facing a crisis situation which is more difficult to manage and probably much more expensive.

The risk of disease transmission in continental Europe related to the presence of *Ae. albopictus* is no longer theoretical [59]. In fact, in the recent past a chikungunya outbreak occurred in Italy in 2007 [60], two indigenous cases were reported in metropolitan France in 2010 [61] and recently four cases of CHK locally-acquired infection in Montpellier, France were notified to WHO [62]. Similarly, autochthonous cases of dengue were described in metropolitan France [63] and Croatia [64,65]. Therefore, the European Centre for Disease Prevention and control has published detailed guidelines for the surveillance of the vector [35]. In Canton Ticino, an average of around 110 eggs/ovitraps were observed during the peak of activity. This number is far lower than the one considered, i.e. > 200 eggs/ovitrap, as the threshold for epidemic risk of chikungunya or dengue [66]. However, an action plan in case of autochthonous cases has been implemented by the GLZ in collaboration with the medical authorities of the Canton Ticino based on the one set in Emilia-Romagna (Italy) [58]. No case of local transmission has been reported so far in Ticino and no infected mosquito has been detected [25].

## Conclusions

To conclude, the objectives we had in 2000 regarding nuisance, sanitary risk and control of the use of insecticides to minimize impact on the environment, are so far, thirteen years later, accomplished. In addition today authorities are prepared to face possible emergencies in a competent way based on experience and up-to-dated information. The *Ae. albopictus* surveillance in Ticino settled also the basis for the elaboration and implementation of the current extended surveillance at the national level in Switzerland [49].
